# Searching in Sediments for Ancient Human DNA

**DOI:** 10.1021/acscentsci.2c00769

**Published:** 2022-07-11

**Authors:** Rachel Brazil

Fragments of stone tools and occasional skeletal remains provide most of what we know about our cave-dwelling human ancestors and their Neanderthal cousins. But in the past 15 years, archaeologists and anthropologists have gained a powerful tool in the next-generation DNA sequencing techniques that have revolutionized the biosciences. Fast, cheap, and accurate sequencing has allowed them to analyze ancient DNA from bones and other sources and expand our understanding of human evolution and migration.

**Figure:**
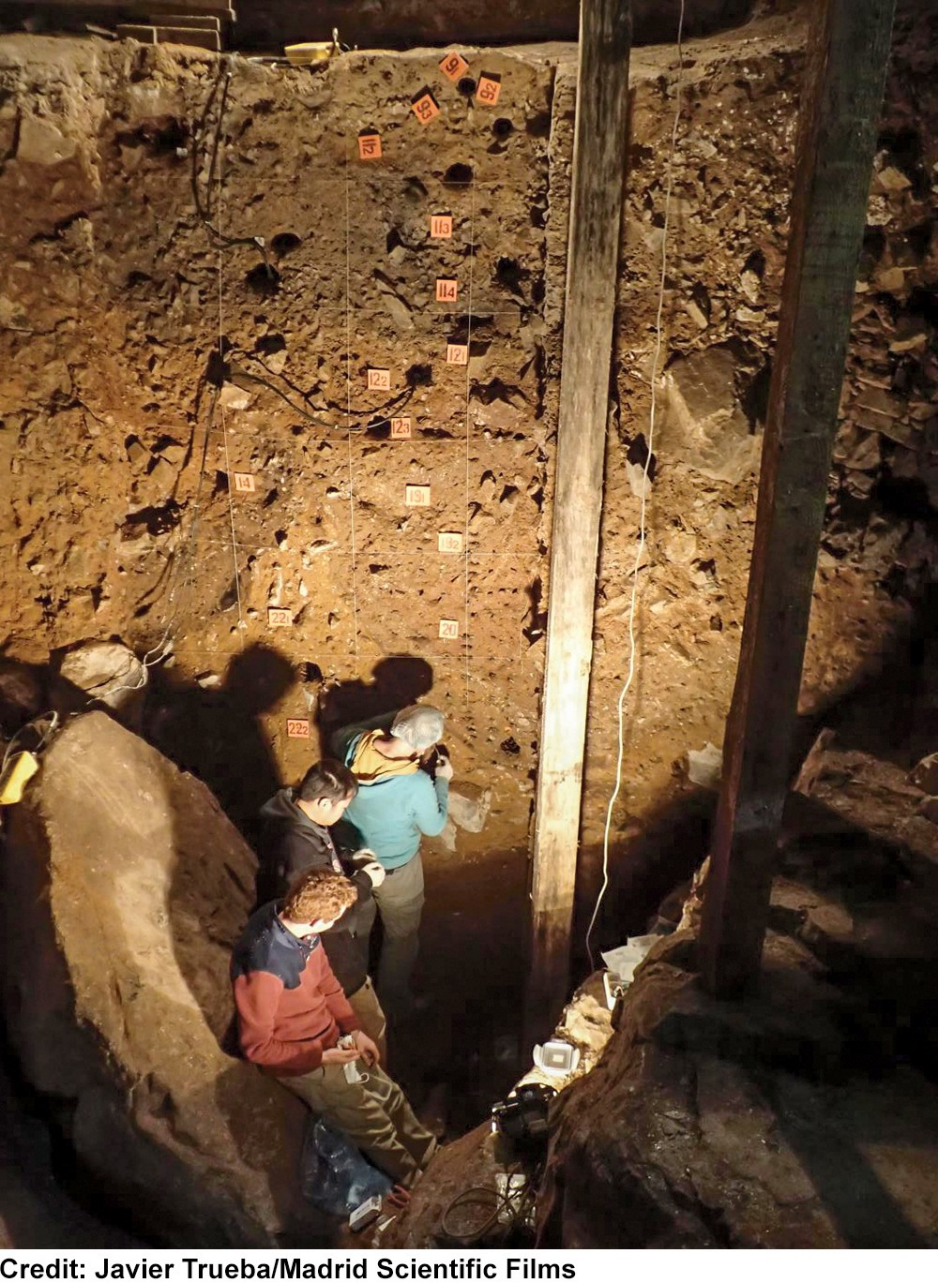
Researchers from the Max Planck Institute for Evolutionary Anthropology collect sediments from the Galeria de las Estatuas cave site in northern Spain.

To date, thousands of archaic human genomes have been sequenced. The oldest sequenced hominin genome goes back 430,000 years, to the last ice age, in the Pleistocene era. During that time, our ancestors coexisted and mated with Neanderthals and Denisovans—the latter a previously unknown group of archaic humans. They were discovered from ancient DNA extracted from a finger bone found inside the Denisova Cave in the Altai Mountains in Siberia in 2008.

Even small fragments of bones and teeth can yield DNA, but such fossils are rare. So, 5 years ago, a team from the Max Planck Institute for Evolutionary Anthropology tried looking not for fossilized bones to sample but for the DNA itself, perhaps left behind from decomposed remains or bodily fluids. That team reported that ancient hominin DNA could be found in soils and sediments in a number of cave sites known to have been occupied across Europe. The researchers extracted and amplified millions of short stretches of Neanderthal and Denisovan mitochondrial DNA from sediment samples ranging from 14,000 to 550,000 years ago.

Although a pioneering paper in 2003 had shown it was possible to find Pleistocene-era DNA in milligram sediment samples, no one before the Max Planck researchers had looked for DNA from ancient humans.

**Figure:**
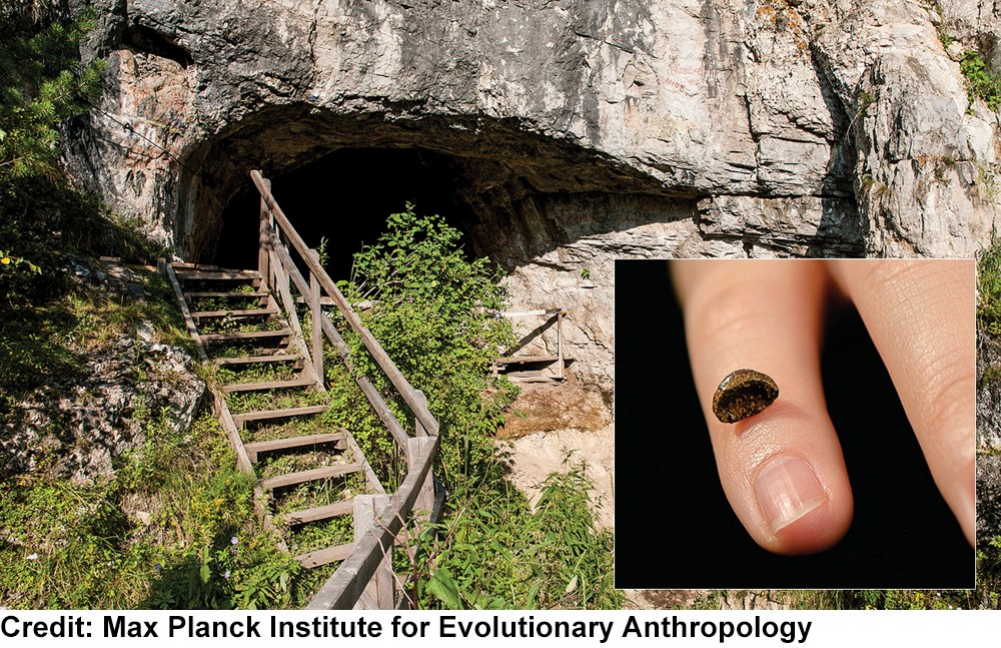
Sequencing DNA from a pinky finger bone (inset) found in the Denisova Cave in Siberia, Russia, revealed a new archaic hominin species.

Several research teams are now looking at which types of sediments may provide the best hunting ground for ancient DNA and how to maximize DNA yields from such sources. With this additional evidence, the researchers hope to better understand the relationship between archaic human groups and their differences to modern humans, and to eventually construct a picture of their migration and population of the world.

## Secrets in the soil

As with DNA extracted from bones or teeth, the material in sediment samples can be “shotgun sequenced,” an approach in which all the DNA present is recovered and sequenced, and the data then pieced together to create larger sections of the genome. But “a vast majority of the DNA we extract is actually microbial—99% or more,” says Matthias Meyer, an evolutionary geneticist who led the team at Max Planck. This means that most of the sequenced DNA is not relevant and makes a shotgun sequencing approach “super costly,” says archaeological scientist Tyler Murchie, a postdoctoral fellow at McMaster University’s Ancient DNA Centre.

Instead, most groups use hybridization capture, a technique that employs predesigned oligomer probes to bind DNA of interest. The probes are attached to magnetic biotin molecules, a move that allows the tagged DNA molecules to be pulled out for sequencing. “It doesn’t have to be a 100% match; there is some wiggle room,” says Viviane Slon, who was a graduate student in Meyer’s lab at Max Planck at the time of the 2008 study and is setting up her own ancient DNA laboratory at Tel Aviv University.

Hybridization capture still lets researchers pick up mutations or differences between sequences characteristic of various archaic humans and of other animal species. The key, Slon says, is choosing probes that target specific parts of the genome that are informative but distinct between humans and Neanderthals. “It’s an incredibly powerful technique” that has generated nearly all of the data on ancient hominins in recent years, Meyer says.

The main challenge in sequencing ancient DNA is the length of the fragments found. Over time, DNA molecules will gradually break down via a number of mechanisms, including hydrolysis. Commercial sequencing kits are designed for extracting modern DNA samples, which start off largely intact. The kits are “not adapted to retrieving such short fragments,” Slon says.

So researchers have developed bespoke protocols using buffers, proteases, and chaotropic salts that will break down cellular matter while preserving as much DNA as possible, as well as compounds that bind inorganic material. These steps remove the sorts of contaminants found with ancient DNA samples, and the strands can be precipitated out by adding alcohol. Meyer says his group has found that adding more alcohol while precipitating out the DNA seems to help recover more of the shorter fragments.

Many ancient DNA laboratories also heat the recovered DNA to separate the strands, which recovers 10 times as much DNA—another innovation from Meyer’s lab. The DNA is subsequently captured on silica beads and prepared for sequencing following standard methods.

Then things get tricky again. Chemical changes that may have occurred to the DNA bases over time affect how the sequences are read. “When you’re analyzing ancient DNA, you have to be aware that you have certain types of mutations that are not real,” says Beth Shapiro, an evolutionary molecular biologist at the University of California, Santa Cruz. For example, at the terminal nucleotides of a DNA fragment, deamination of the base cytosine leaves uracil, which sequencers identify as thymine. Some laboratories use the enzyme uracil-DNA glycosylase (UDG) to convert the uracil back to cytosine so that they can distinguish between thymine and cytosine in damaged DNA. Others detangle the data by comparing it with reference sequences.

Shapiro’s lab and others do not use UDG, as they prefer to see cytosine to thymine changes “as proof that it’s authentically old,” Shapiro says. This helps differentiate ancient DNA from any modern human DNA that has tainted the samples. To limit such contamination, researchers always extract the DNA under clean-room conditions, but the tiny amount of ancient DNA in any sample means that just one modern human cell can swamp the ancient DNA within.

Early results provided sequences only from mitochondrial DNA, which is easier to find because each cell has hundreds of copies, compared with one copy of the nuclear genome. But because it is inherited from the mother, mitochondrial DNA provides limited potential for understanding population differences. Nuclear DNA “gives us the full picture,” Meyer says. In May 2021, his group published the first ancient human nuclear DNA sequenced from cave sediments, dating from between 50,000 and 200,000 years ago. The researchers could identify two distinct Neanderthal populations inhabiting the cave, located in northern Spain’s Galería de las Estatuas, one having replaced the other 100,000 years ago.

## Stuck on sediments

These sediment analysis techniques offer a major new opportunity to study ancient human DNA and to identify places where archaeologists and anthropologists can make discoveries. Most data currently come from permafrost regions, where DNA survives longer. DNA preservation in temperate climates is limited to around 100,000 years, Meyer says, and in the tropics, “it’s rarely possible to get something that’s older than about 10,000 years.” He hopes that screening sediments in Africa or the Middle East may start to reveal sites in those regions with preserved DNA that could push those limits to broaden the picture of human evolution and where and how our ancestors lived.

But, Meyer says, “we don’t quite understand how the DNA gets there and how it is preserved over time.” One possibility is through binding to sediment materials, which he and his group have tested by adding highly concentrated DNA to clay. Much of the DNA gets bound to the clay and cannot easily be washed off again with water.

Chemist Colin Freeman of the University of Sheffield and collaborators at the University of Copenhagen have also been investigating how DNA may be preserved on sediment. DNA’s phosphate groups give it a negative charge, and so it is adsorbed on top of a tightly bound water layer on mineral surfaces through ion bridges that balance the charge. Freeman and colleagues have studied DNA interactions with calcium carbonate (calcite) surfaces and have observed some direct bonding. “The calcite surface naturally forms little steps as it grows, and it preferentially binds [DNA] on the edges of those steps,” Freeman says. But the researchers have yet to discover if any one binding mechanism predominates with the ancient DNA preserved in sediments.

Mineral surfaces may protect adsorbed DNA molecules from hydrolysis reactions by hindering access to reactive sites. It is also possible the mineral grows around the DNA molecule, encapsulating it. Ron Pinhasi of the University of Vienna and his colleagues found ancient mammalian and plant DNA preserved within the layers of stalagmites that were deposited between 56,000 and 84,000 years ago in caves in eastern Europe.

The length of DNA strands recovered from sediments does not differ greatly from those found in fossils, but retrieving the DNA is more difficult. “There’s a whole bunch of other stuff in the sample compared to, say, bone,” Murchie says. “In particular, humic acids can be challenging.” Humic acids are a complex mixture of long-chain molecules resulting from the decomposition of biological matter. These additional substances can inhibit the enzymatic reactions needed to sequence DNA. But removing humic acids with harsh reagents will damage the DNA. It is a balancing act “of trying to maximize our DNA recovery while minimizing the kind of corecovery of other stuff that we don’t want,” Murchie says.

**Figure:**
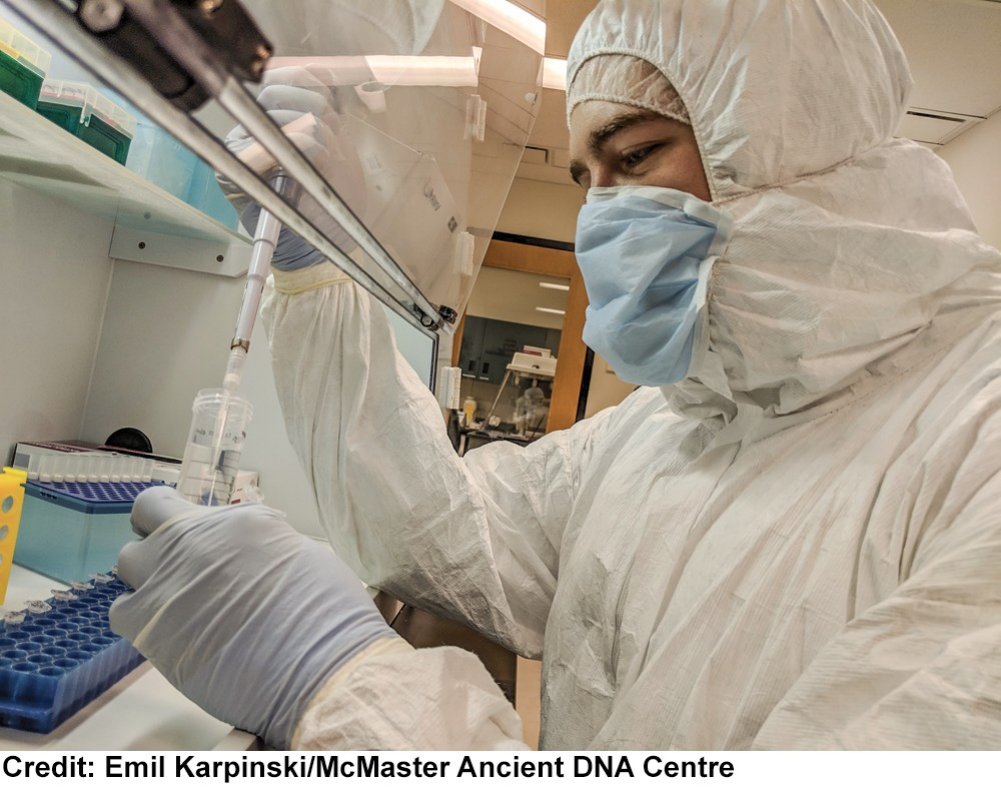
Tyler Murchie of McMaster University conducts an experiment on ancient DNA under clean-room conditions to prevent sample contamination. (Eye protection is not required for the work in this laboratory, according to an institutional risk assessment.)

The method Murchie and colleagues have found to be most successful is spinning their samples at 4 °C to precipitate out unwanted material, which allows 8–19 times as much DNA to be recovered as with commercial extraction kits. Murchie used the method to find 11,000-year-old woolly mammoth DNA in just a few grams of soil from the Yukon. But he admits that there are still difficulties identifying and removing compounds that inhibit DNA extraction for about one in five sediments they encounter, and they have been unable to identify the problematic molecules from mass spectrum analysis. “We just need to get some chemists involved to really help us out,” he says.

Pinhasi became curious as to whether certain sediments were more likely than others to preserve DNA after his team shotgun sequenced a single 25,000-year-old sediment sample from a cave in western Georgia and found a surprising level of diversity: human, wolf, and bison DNA, all in relatively large quantities. “Nobody so far has managed to get anything remotely close to the amount of DNA that we have in this one sediment,” he says. But “we have no idea why.”

Pinhasi is now collaborating with University of Vienna environmental geochemist Stephan Kraemer to look at real and model sediments to determine what types might best preserve DNA. “We really want to understand a bit more” before continuing to randomly test sediment samples because of the high cost of sequencing, Pinhasi says. “Then we’ll come to the tricky part of [developing] the best mechanisms or protocols to separate” the DNA.

Kraemer says that manganese oxides, for example, might actually catalyze DNA destruction under certain conditions, while other minerals might help to preserve it.

“I am still just amazed, almost on a daily basis, by the fact that we can recover Neanderthal, Denisovan, and human DNA from sediments,” Meyer says. He often works with archaeologists who have spent years excavating sites with an abundance of stone tools but seemingly no trace of the individuals who made them. Now he is often able to tell them who those ancient humans were.

*Rachel Brazil is a freelance contributor to**Chemical & Engineering News**, an independent news outlet of the American Chemical Society.*

